# Mg-Ca-Sr Biodegradable Alloys for Medical Applications: Production, Biomaterials’ Properties Characterization, and In Vitro and In Vivo Biocompatibility Evaluation

**DOI:** 10.3390/bioengineering12090939

**Published:** 2025-08-30

**Authors:** Gabriela Leață, Kamel Earar, Corneliu Munteanu, Fabian Cezar Lupu, Maria Daniela Vlad, Bogdan Istrate, Ramona Cimpoesu, Aurelian-Sorin Pașca, Eusebiu Viorel Șindilar

**Affiliations:** 1 Faculty of Dental Medicine, “Dunarea de Jos” University, 800008 Galati, Romania; gabrielaleata@yahoo.com (G.L.); kamel.earar@ugal.ro (K.E.); 2 Mechanical Engineering, Mechatronics and Robotics Department, “Gheorghe Asachi” Technical University of Iasi, 700050 Iasi, Romania; corneliu.munteanu@academic.tuiasi.ro (C.M.); bogdan.istrate@academic.tuiasi.ro (B.I.); 3 Technical Academy of Sciences Romania, 010413 Bucharest, Romania; 4 Faculty of Medical Bioengineering, “Grigore T. Popa” University of Medicine and Pharmacy from Iaşi, 700115 Iasi, Romania; 5 TRANSCEND Research Centre, Regional Institute of Oncology, 700483 Iasi, Romania; 6 Faculty of Materials Science and Engineering, The “Gh. Asachi” Technical University from Iaşi, 700050 Iaşi, Romania; ramona.cimpoesu@academic.tuiasi.ro; 7 Faculty of Veterinary Medicine, Iasi University of Life Sciences “Ion Ionescu de la Brad”, 700490 Iasi, Romania; sorin.pasca@iuls.ro (A.-S.P.); eusebiu.sindilar@iuls.ro (E.V.Ș.)

**Keywords:** biomaterials, magnesium alloy, microstructural analysis, corrosion analysis, in vivo tests, in vitro tests

## Abstract

The research of biomaterials is an area of significant interest in the biomedical field, and the present study investigates how the strontium (Sr) concentration influences the microstructure, corrosion resistance, and both in vitro and in vivo behavior of alloys in the ternary Mg-Ca-Sr system. Using an induction furnace with a controlled atmosphere (argon as the shielding gas), Mg-0.5Ca-xSr alloys (x = 0.5; 1; 1.5; 2; 3 at.%) were synthesized. Microstructural analyses, performed using optical microscopy and scanning electron microscopy (SEM), revealed a uniform and refined structure. Corrosion behavior assessments, carried out using linear and cyclic potentiometry, demonstrated favorable corrosion resistance for all samples. However, for the system containing 0.5% Sr, the corrosion rate values were lower compared to the other systems, and this alloy also exhibited the lowest corrosion current density. Cytocompatibility assay indicated the cytocompatible behavior of all the studied alloys, with favorable influence on cell viability and a stimulatory effect on the osteoblastic cell proliferation. In vivo biocompatibility assessments of the alloys showed that, for alloys containing 0.5% and 1% Sr, a more rapid degradation occurred in comparison with the other alloys (1.5, 2 and 3% Sr), which still persisted at the tissue level even after 12 weeks post-implantation. In all the batches examined, the inflammatory reaction was directly proportional and persistent in relation to the presence of the material in the tissue. In regions where the material was resorbed/degraded, the local inflammatory response was reduced or absent, and the fibrous tissue was denser and better organized. The field of biomaterials is in continuous development, and this study highlighted the applicability of these five alloy systems for dental and maxillofacial applications such as implants, plates, and related devices.

## 1. Introduction

**Figure 1:**
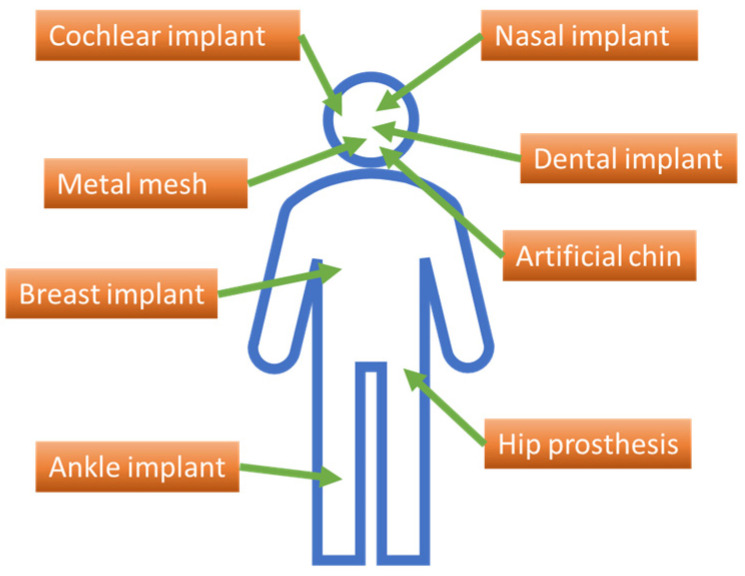
Various applications of biomaterials in the human body.

Biomaterials represent a continuously developing field [1] due to their major impact in the medical area, as evidenced by their wide range of practical applications (Figure 1 and Table 1). The successful use of a biomaterial depends on a series of factors, including the properties and biocompatibility of the materials employed, clinically determined cytotoxicity, the necessity of implantation according to the patient’s needs, and the surgeon’s decision regarding the selection of material and personalized design. Table 1 presents the advantages, disadvantages, and several examples of medical applications of the main types of synthetic biomaterials [2,3,4].

In the medical context, these materials are used for various therapeutic purposes, including the replacement of anatomical structures, supporting tissue regeneration and healing, restoring or improving physiological functions, and correcting diverse dysfunctions or malformations [1].

Some medical devices—such as orthopedic implants or those used in maxillofacial surgery, miniplates, screws, membranes, and metallic meshes employed in procedures of osteosynthesis, ligament stabilization, or bone augmentation—are manufactured from biomaterials, which are materials compatible with human tissues [5,6,7].

An emerging research direction [8,9,10] involves the use of biodegradable biomaterials in the design of these implants, membranes, and meshes, offering considerable benefits for both the patient and the clinician. These biodegradable structures are designed to support processes of tissue regeneration and healing, with the intention of being fully absorbed by the organism once their therapeutic function has been fulfilled, by virtue of controlled biocompatibility and biodegradability properties.

Magnesium, a naturally occurring element in the human body, is used as a biodegradable material for orthopedic implants due to its ability to undergo complete degradation without inducing local or systemic toxic effects. However, its use presents certain limitations related to its lower mechanical strength compared to non-resorbable metallic implants, a high corrosion rate that may surpass the rate of bone regeneration, the release of hydrogen gas with the potential for its accumulation in peri-implant tissues and the associated risk of necrosis or embolism, alterations in serum magnesium concentration due to excessively rapid degradation, as well as a potential hemolytic effect, given that the hemolysis rate for pure magnesium (25–75%) may exceed the threshold considered safe (30%) [11,12,13].

In the process of producing magnesium alloys used in metallurgical and biomedical fields, elements such as calcium (Ca), zinc (Zn), manganese (Mn), zirconium (Zr), and rare earth elements (RE) are frequently added, each playing specific roles in enhancing the final properties of the material. The most notable among these are calcium and zinc, which optimize the mechanical characteristics, and manganese, used as a secondary element for rafination through the formation of intermetallic phases. Calcium and zirconium, at low concentrations, which contribute to increased mechanical strength. Additionally, alloys containing rare earth elements, especially those including gadolinium (Gd), offer superior corrosion resistance and allow for controlled biodegradability in correlation with the processes of resorption and bone apposition. Examples include compositions such as AZ91, AZ31, AE21, LAE442 or WE43—the latter containing yttrium (Y), zirconium (Zr), and rare earth elements—designed to increase thermal stability and creep resistance. As long as the alloying elements remain in the formed solid solution, they can be used to strengthen it. Moreover, most of the aforementioned alloying elements react with magnesium or with each other to form intermetallic compounds. These compounds contribute to the increased strength of the alloy through precipitation strengthening [14,15,16,17].

Magnesium (Mg) and its alloys—particularly those enriched with calcium—represent a promising option in the development of biodegradable biomaterials, given that they are composed of elements naturally present in the human body, such as Mg, Ca, Si, and Sr, the latter being introduced in the present study [18,19,20].

The addition of calcium (Ca) and strontium (Sr) as alloying elements in magnesium leads to the precipitation of the intermetallic phases Mg_2_Ca and Mg_17_Sr_2_ at the grain boundaries, contributing to microstructural refinement and the improvement of the alloy’s mechanical strength. However, these phases exhibit increased brittleness, making the material more susceptible to cracking and negatively affecting its ductility, while their presence also promotes accelerated degradation through galvanic corrosion mechanisms [21,22,23].

Calcium significantly contributes to the strengthening of the solid solution and the precipitated phases, while also acting, to a certain extent, as a structural refining agent by reducing grain size and reinforcing grain boundaries. In parallel, zirconium stands out as an effective grain refiner in aluminum-free magnesium alloys, with the Hall–Petch effect explaining the structural strengthening achieved through the formation of fine grains and the corresponding reinforcement of their boundaries [24,25,26].

In vitro evaluations conducted on cell lines such as MG-63 and SaOS-2 have demonstrated increased cellular tolerance for Mg-Sr alloys compared to pure magnesium. Specific assays (MTT, LDH) indicated sustained cellular activity and efficient proliferation, accompanied by low levels of cytotoxicity, as supported by higher IC50 values [27,28].

In vivo studies conducted on animal models (e.g., rats and rabbits) have demonstrated harmonious integration of the implants, with accelerated bone regeneration and minimal inflammatory reactions. Peri-implant histological analyses did not reveal necrosis, but rather a well-structured extracellular matrix, indicating high cellular viability around the implanted material [29,30].

In the context of using magnesium alloys in medical applications, the selection of alloying elements should not be based solely on their ability to improve mechanical properties and corrosion behavior, but must also take into account their degree of biocompatibility. Once released into the body through biodegradation processes, these elements must not be toxic, either locally or systemically [31,32].

Incorporating calcium and strontium into magnesium-based alloys is particularly interesting for skeletal and maxillofacial applications. As an essential component of bone, calcium enhances biocompatibility and supports mineralization and also Mg-Ca pins demonstrate gradual in vivo degradation and significant osteogenesis by the third month [33]. Strontium stimulates osteoblastic activity while inhibiting osteoclast-mediated resorption, thereby promoting bone regeneration. Furthermore, strontium has been reported to contribute to improved corrosion resistance at moderate concentrations. The studied alloys in the Mg-Ca-Sr ternary system (as biomaterials that gradually degraded/resorbed in the body as the bone heals, and with the advantage of avoiding the stress shielding phenomenon) are therefore promising candidates for temporary orthopedic and/or maxillofacial implants. Understanding its corrosion and in vitro and in vivo behavior is therefore critical for its future clinical application.

## 2. Materials and Methods

### 2.1. Mg-Ca-Sr Alloys Fabrication

**Figure 2:**
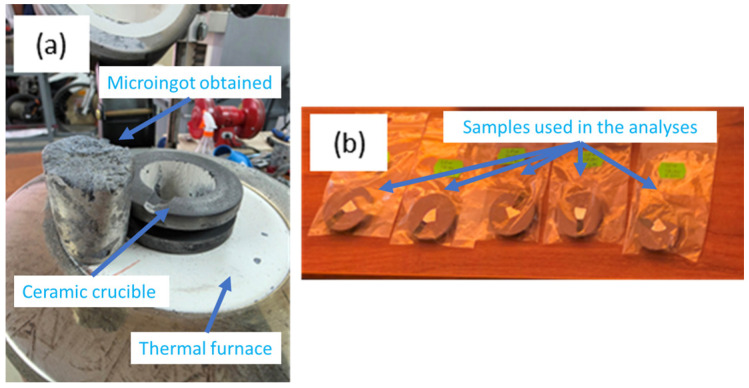
Example of ingot obtained after casting (**a**) and specimens for analysis (**b**).

In the casting process of alloys from the Mg-Ca-Sr system (Mg-0.5Ca-xSr; x = 0.5; 1; 1.5; 2; 3%), using the REX C100 thermal furnace (RKC Instruments, Inc., Tokyo, Japan), the materials were melted in ceramic crucibles, under a controlled argon (Ar) atmosphere to avoid the risk of oxidation or flammability of the alloy. In order to obtain the specific chemical compositions, specific batch calculations were performed and high-purity principal elements were used. The samples were designated as follows: Sample G1 = Mg-0.5Ca-0.5Sr; Sample G2 = Mg-0.5Ca-1Sr; Sample G3 = Mg-0.5Ca-1.5Sr; Sample G4 = Mg-0.5Ca-2Sr; Sample G5 = Mg-0.5Ca-3Sr. After the casting process, the resulting samples underwent a homogenization heat treatment at 420 °C for 12 h, followed by slow cooling together with the furnace. Figure 2 presents the ingots obtained after casting as well as the experimental specimens produced from these ingots.

The Sr content was varied in the range of 0.5 to 3 wt.% in order to investigate the effect of both low and high concentrations on the corrosion behavior and microstructure of Mg-0.5Ca-xSr alloys.

### 2.2. Analysis of Microstructure, Chemical Composition and Corrosion Properties

After obtaining the ingots, for their sectioning operations, a Metacut 302 cutting machine (Metkon, Bursa, Turkey) was used.

The samples were embedded in resin using the Ecopress 52 (Metkon, Bursa, Turkey), and the surface grinding operations were performed with a Forcipol 202 (Metkon, Bursa, Turkey). After these steps, metallographic etching was carried out so that the samples could be used for microstructural analyses.

Microstructural analyses by optical microscopy were performed using a Leica DMI5000 M microscope (Leica, Wetzlar and Mannheim, Germany). Microstructural analyses by electron microscopy and EDS (Elemental Distribution Surface) analyses were carried out with a Thermo Scientific Quattro C microscope (Brno, Czech Republic). Higher magnifications were used due to the refined structure.

For X-ray diffraction (XRD) investigations, the X’Pert PRO MPD X-ray diffractometer (Panalytical, Almelo, The Netherlands) was used. For electrochemical corrosion testing, the Origa Flex potentiostat produced by OrigaLys ElectroChem SAS (Rillieux la Pape, France) was utilized.

To evaluate the corrosion behavior, open circuit potential (OCP) measurements were performed, followed by potentiodynamic polarization tests to determine characteristic corrosion parameters such as corrosion potential and corrosion current [34,35]. The tests were carried out in a simulated body fluid (SBF) electrolyte, prepared in the laboratory according to Kokubo et al. [14], with its chemical composition presented in Table 2.

For the electrochemical experiments, a three-electrode cell equipped with a controlled stirring system was used. In these experiments, a platinum electrode served as the auxiliary electrode, while a saturated calomel electrode (SCE) was used as the reference electrode. Electrochemical measurements were performed at a controlled temperature of 25 °C, under natural aeration of the electrolyte. Linear polarization curves were recorded at a potential scan rate of 1 mV/s, within an interval of ±150 mV with respect to the open circuit potential (OCP). For extended electrochemical characterization, cyclic polarization curves were obtained at a scan rate of 10 mV/s, within the potential range of −700 mV to +1000 mV.

The following parameters were determined: the corrosion potential corresponding to zero corrosion current E (I = 0), the anodic and cathodic Tafel slopes (ba, −bc), polarization resistance (Rp), corrosion current density (jcorr), and the associated corrosion rate (vcorr). The surface morphology of the samples, before and after the electrochemical treatment, was examined using a scanning electron microscope (SEM), in order to highlight microstructural changes associated with the corrosion process.

For this study, magnesium-based alloy samples with the chemical compositions Mg-0.5Ca-xSr (x = 0.5; 1; 1.5; 2; 3%) were used. The samples were sectioned, followed by progressive mechanical grinding up to P2400 grit size and final polishing with an alumina suspension (0.05 μm) to ensure a reproducible surface.

Immersion tests were performed in simulated body fluid (SBF), prepared according to the composition proposed by Kokubo, which reproduces the ionic conditions of human plasma. The solution was buffered at a pH of 7.4 and maintained at a constant temperature of 37 ± 0.5 °C in a thermostated enclosure, to simulate physiological conditions.

Each sample was fully immersed in 50 mL of SBF in a sealed polypropylene vessel, covered to minimize evaporation. The duration of the test was 72 h (3 days), under static conditions, with periodic monitoring of the solution’s pH. At the end of the immersion period, the samples were removed, rinsed with distilled water and ethanol, then dried in a controlled atmosphere. The corrosion rate was determined gravimetrically, by measuring the mass loss after ultrasonic removal of corrosion products. Morphological changes in the sample surfaces, before and after the immersion test, were analyzed using a scanning electron microscope, and the chemical composition of the corrosion layers was investigated by energy-dispersive spectroscopy (EDS).

### 2.3. Cytocompatibility Assay

#### 2.3.1. Alloy Extracts Preparation

The samples corresponding to the five alloy systems, in the form of flat metallic specimens with well-defined mass, were cleaned with ethanol and exposed to UV for 30 min on each side. Afterwards, they were immersed for 48 h in sterile, complete MEM culture medium at pH 7, at an extraction ratio of 0.1 g/mL (according to ISO-10993-12 and ISO-10993-5 standards; [36,37]), in order to obtain extracts to be used in various dilutions (i.e., extract 10%, 20%, 40%, 60%, 80%, 100% per well) for cytotoxicity assessment. The prepared samples were kept under sterile conditions to obtain the extracts.

#### 2.3.2. Cell Culture

MG-63 cells (a commercial cell line isolated from human osteosarcoma) were selected for their osteoblastic phenotype, being extensively used for cytotoxicity tests. The cells were cultured in Minimal Eagle Medium (MEM; Sigma-Aldrich, St. Louis, MO, USA) supplemented with 10% decomplemented fetal bovine serum (FBS; Sigma-Aldrich, St. Louis, MO, USA), 2% L-glutamine, and 1% antibiotic (penicillin-streptomycin) in standard conditions (at 37 °C, 5% CO_2_ and high relative humidity > 95%) until confluence. Then after, the culture medium was removed, the adherent cells were washed with PBS (phosphate-buffered saline solution; Sigma-Aldrich, St. Louis, MO, USA), detached with trypsin-EDTA, centrifuged, counted and seeded onto 48-well culture plates at a density of 10^4^ cells/well.

#### 2.3.3. Cell Viability Testing

Cell viability was tested by the MTT assay (using a tetrazolium salt solution; 3-[4,5-dimethylthiazol-2-yl]-2,5-diphenyltetrazolium bromide; as was published by Vlad et. al [38,39,40]) which allows quantification of the number of living cells (that convert the pale-yellow solution into a blue intracellular formazan crystal, by the contribution of mitochondrial enzymes). Briefly, the cells seeded onto 48-well culture plates were cultured for 24 h, then the media was changed by the alloy extracts’ dilutions (as was mentioned in the above section) and co-incubated with the alloys’ extracts for 1 and 3 days. After the specified time intervals, the liquid from the wells was replaced by the MTT solution and incubated for 3 h; then the solution was removed and the resulting formazan crystals were solubilized with dimethyl sulfoxide (DMSO) followed by the quantification of the color intensity at a wavelength of 570 nm, using a spectrophotometer (FilterMax F5 Multimode Microplate Reader; Molecular Devices, San Jose, CA, USA).

Cell viability was expressed as a percentage relative to control wells, using the formula: V (%) = 100 × (As/Ac), where As is the absorbance for the sample, and Ac is the absorbance for the control wells (cells cultured only with cell culture medium, without any extract added). The viability assay was performed in triplicate.

Statistical analysis of the cell viability results was performed using the one-way ANOVA test, and the data were compared by the Tukey method, with statistical significance being accepted for *p* < 0.05.

#### 2.3.4. Cell Morphology Evaluation

After MG63 cells co-incubating with the 100% alloys’ extracts for 3 days (under the previously specified conditions), the cell morphology study was performed by fluorescence microscopy after fluorescent labeling of viable cells with Calcein-AM. For this, after the specified time, the cells were washed with HBSS (Hanks’ Balanced Salt Solution) without phenol red, and then 100 μL of a 1:1000 calcein solution in HBSS was added to each well and the plate was incubated in the dark for 30 min. Subsequently, cell morphology was evaluated using an inverted fluorescence microscope (Leica DMIL LED, equipped with Leica DFC450C camera and image acquisition software—Leica Application Suite—Version 7.4.1; Leica, Wetzlar, Germany).

### 2.4. Conducting In Vivo Analyses

For the in vivo tests, the use of animals was required, thereby imposing both scientific and ethical rigor. The study complied with the ethical directives presented in the Guide for the Care and Use of Laboratory Animals (Law 43/2014), the European legislation regarding animal use (EU/2010/63-CE86/609/EEC), and the animal experiments were approved by the Ethics Committee of the Faculty of Veterinary Medicine, “Ion Ionescu de la Brad” University of Life Sciences in Iași, Romania (Decision No. 1791/27/12/2024).

#### 2.4.1. Implantation

The selection of laboratory animals was designed with the principle of minimizing animal use while maintaining scientific rigor. To achieve this, two implants were placed in the same rat—either in both pelvic limbs or in one pelvic limb and the lumbar region. This approach ensured that the number of animals used was kept to a minimum while still meeting the experimental needs.

Seventy-five male Wistar rats, aged approximately 30 to 32 weeks, with a body weight of 350 ± 15 g, were randomly assigned to five groups corresponding to five different concentrations used in the study.

Prior to the experiment, the rats underwent an acclimatization period of 5 days in the laboratory. They were housed under controlled conditions: a temperature of 22 ± 0.7 °C, humidity of 60 ± 10%, and a 12 h light/dark cycle. During this time, the rats were monitored daily for signs of stress or abnormal behavior due to transport or environmental adjustment.

The rats were purchased from the Cantacuzino National Research and Development Institute for Microbiology and Immunology in Bucharest, Romania. At the femoral region, two pieces of each alloy were implanted. The alloys, composed of Mg-Ca-0.5% with varying amounts of strontium (0.5%, 1%, 1.5%, 2%, 3% by weight), were obtained in cylindrical bar ingot form and then cut into small samples for implantation.

Implants were designed with rounded corners and had a parallelepiped shape, measuring 10.4–11.1 mm in length, 4.7–5.2 mm in width, and 2.1–2.2 mm in height. The evaluation of soft tissue reactions and biodegradation of the implants was conducted at 1, 4, and 8 weeks post-implantation.

#### 2.4.2. Pre-Operative Preparations and Surgical Method

Rats underwent surgery under general anesthesia. The anesthetic protocol involved intramuscular injection of xylazine and ketamine, followed by maintenance of anesthesia using an inhaled mixture of oxygen and 1–2% isoflurane. Once anesthesia was induced, the rats were placed in a sternoabdominal recumbency on the surgical table, and the surgical site was disinfected with a betadine solution.

Two anatomical regions were selected for implantation to reflect potential human applications in spine and long bone pathology, such as fractures. Two implants were placed in each rat—either in both pelvic limbs or in one pelvic limb and the vertebral lumbar region. This strategy ensured that no animal life was unnecessarily sacrificed.

**Figure 3:**
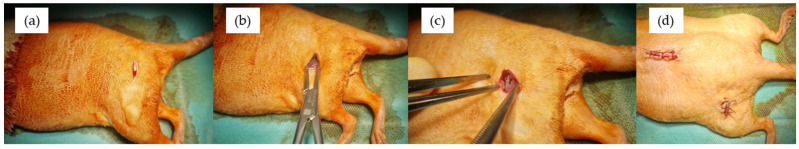
Images from the surgical procedures: step 1 (**a**); step 2 (**b**); step 3 (**c**); step 4 (**d**).

The surgical procedure involved the following steps (refer to Figure 3):

1. Incision of the skin and fascia;

2. Muscle dissection and creation of a periosteal and cortical defect in the femoral diaphysis and vertebral arch in the lumbar spine region;

3. Implantation of the alloys, ensuring direct contact with bone tissue, periosteum, and surrounding musculature;

4. Tissue reconstruction, including suturing of the musculature using continuous 2/0 PGA sutures and skin closure with separate point stitches.

Post-operative, the rats were allowed to move freely within their cages and were closely monitored for any visual signs of mobility issues or other complications. No adverse effects were observed during the recovery period.

#### 2.4.3. Histological Processing

For the collection of samples for histopathological examination, rats from all experimental groups were euthanized using isoflurane, at the time points specified in the experimental protocol, namely 2 weeks (labeled A), 4 weeks (B), and 12 weeks (C). All harvested specimens were processed using the paraffin embedding technique.

Immediately after euthanasia, tissue fragments containing the implanted materials were collected from each animal for histopathological examination and immersed in 10% buffered formaldehyde for 48 h for fixation. The collected specimens were then subjected to demineralization in 10% trichloroacetic acid for 5 days, with daily solution replacement. Paraffin embedding was performed using a Leica TP1020 tissue processor (Leica Microsystems GmbH, Germany). Sectioning was conducted at a thickness of 5 μm using the SLEE CUT 6062 microtome (SLEE Medical GmbH, Wetzlar, Germany).

After deparaffinization, the sections were stained using the Masson’s trichrome technique. Qualitative histological examination was performed using the Leica DM microscope (Leica Microsystems GmbH, Wetzlar, Germany) with a Leica ICC50 HD, 5 MPX, histology camera (Leica Microsystems GmbH, Wetzlar, Germany). Microphotographs were obtained using Leica Application Suite Software (LAS) version 4.2. The entire protocol was carried out under standardized conditions for all tissue samples.

## 3. Results and Discussions

### 3.1. Microstructural Analysis of the Mg-Ca-Sr Alloys

**Figure 4:**
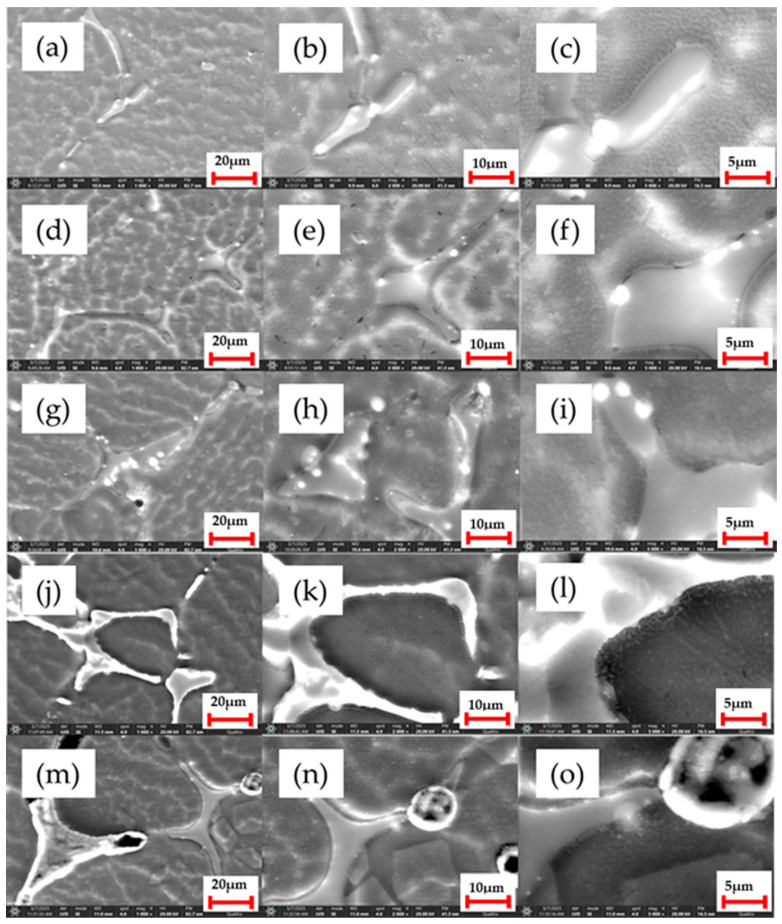
SEM images for the samples: G1 (**a**–**c**); G2 (**d**–**f**); G3 (**g**–**i**); G4 (**j**–**l**); and G5 (**m**–**o**). Magnifications by columns: ×1000, ×2000 and ×5000.

Figure 4 shows the microstructure details of the five studied alloys in the Mg-Ca-Sr systems with varying Sr concentrations (i.e., 0.5; 1; 1.5; 2; 3%).

As can be observed from the SEM image analyses, the samples exhibit a homogeneous, uniform, and refined microstructure without inclusions or other defects. The alloys, showing a uniform distribution of phases, display visible microstructural changes with the increasing Sr content (as seen starting with sample G2), indicating the formation of new phases. In the case of sample G3, it can be seen that, due to the higher Sr content, the microstructure is characterized by a more complex distribution of phases, highlighting zones of interaction between the main phases, which consequently influence the alloy’s properties. For samples G4 and G5, similar details can be noted, and it is observed that increasing the amount of strontium leads to variable phase sizes.

### 3.2. Chemical Composition Analysis of the Mg-Ca-Sr Alloys

**Figure 5:**
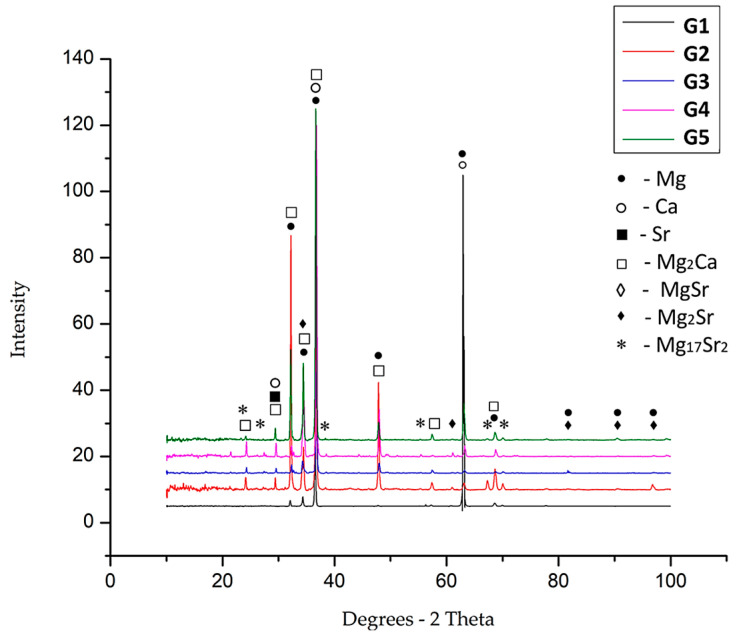
XRD graphs obtained for the five alloy systems: G1—black line; G2—red line; G3—blue line; G4—pink line; and G5—green line.

The identification of the component phases for each experimental alloy was per-formed by X-ray diffraction, an analysis that reveals characteristic diffraction peaks. Chemical compounds such as Mg_2_Ca, MgSr, and Mg_2_Sr could be identified and of course, solid solution elements based on Mg, Ca and Sr. In Figure 5, the graphs for the five alloy systems are presented.

### 3.3. Corrosion Behavior Analysis of the Mg-Ca-Sr Alloy System

The electrochemical corrosion parameters are presented in Table 3.

For the composition with x = 1% Sr (G2), the potential formation of the Mg_17_Sr_2_ intermetallic phase occurs in a controlled manner, being uniformly and finely distributed within the microstructure. This homogeneous distribution helps reduce the risk of localized attack and stabilizes the general corrosion mechanism. In the case of this alloy, the most favorable electrochemical behavior was observed, with a significant reduction in corrosion rate compared to the sample with x = 0.5% Sr, recording values of 4.56 mm/year versus 10.95 mm/year. Additionally, this alloy showed the lowest corrosion current at 0.1981 mA/cm^2^.

**Figure 6:**
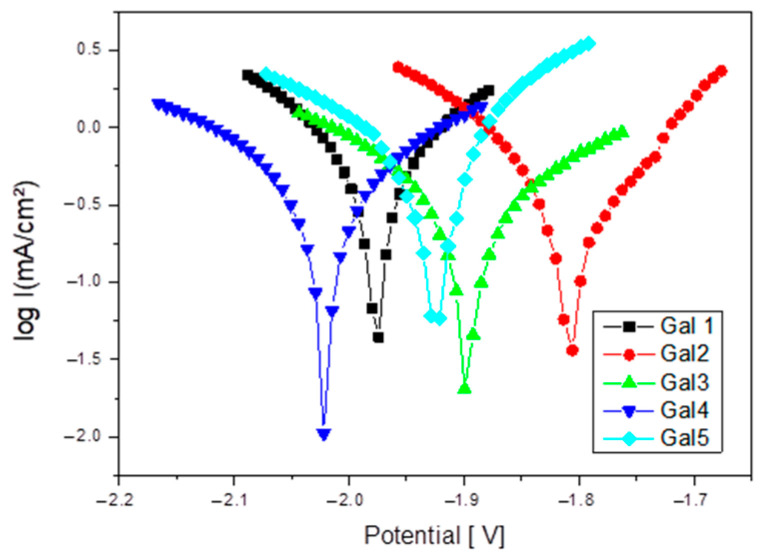
Tafel plots for the Mg-0.5Ca-xSr samples (x = 0.5; 1; 1.5; 2; 3%) in SBF electrolyte solution.

Increasing the strontium content in the Mg-0.5Ca-xSr alloy from 0.5% to 3% results in a non-linear evolution of the corrosion behavior. At low concentrations (0.5–1%), Sr slightly improves corrosion resistance due to the refined microstructure. However, upon exceeding the threshold of ~1.5%, excess Sr leads to the formation of cathodic intermetallic phases (e.g., Mg_17_Sr_2_), which facilitate galvanic corrosion. Thus, the corrosion rate increases significantly at x ≥ 2%, indicating that this content becomes detrimental for applications where corrosion resistance is essential. Figure 6 shows Tafel plot for samples tested in SBF electrolyte solution.

**Figure 7:**
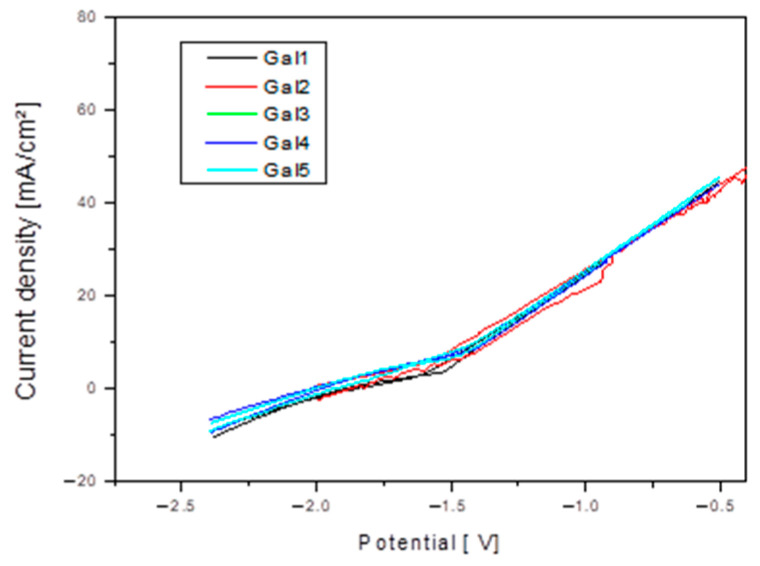
Cyclic polarization plots for the Mg-0.5Ca-xSr samples (x = 0.5; 1; 1.5; 2; 3%) in SBF electrolyte solution.

At x = 2 and 3% Sr (G4 and G5), the corrosion increases significantly compared to the values recorded for alloys with lower Sr content, reaching a corrosion rate value of 14.73 mm/year. This range is considered excessive, leading to the massive formation of Sr-rich cathodic phases and to the acceleration of galvanic corrosion. Figure 7 shows Cyclic polarization plots for samples tested in SBF electrolyte solution.

The corrosion rate, determined gravimetrically by measuring the mass loss after the ultrasonic removal of corrosion products, is shown in Table 4.

The corrosion rate for the Mg-0.5Ca-xSr system shows a significant dependence on the strontium (Sr) content. At a low Sr concentration (x = 0.5%), the corrosion rate value is relatively high, 7.83 mm/year, indicating limited protection provided by the initial Sr addition. A decrease in corrosion rate to 6.98 mm/year is observed at x = 1%, suggesting an optimal composition where the Mg_17_Sr_2_ intermetallic phases are finely and uniformly distributed, reducing susceptibility to localized galvanic attack.

**Figure 8:**
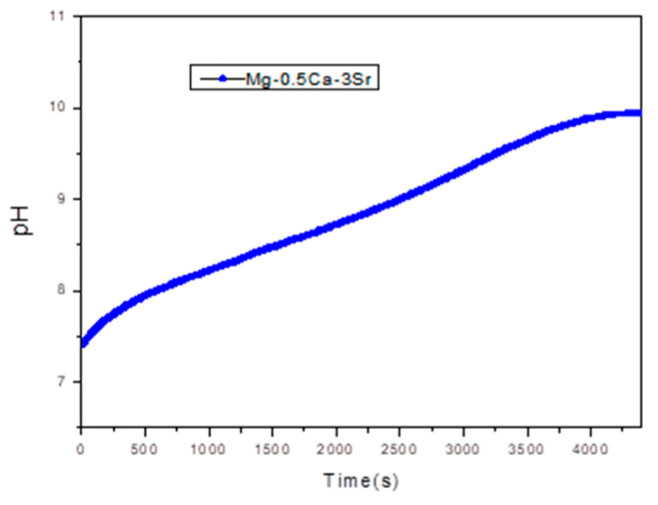
pH variation recorded for sample G5 over 72 h.

At higher Sr contents (x ≥ 1.5%), the corrosion rate increases progressively, reaching 11.81 mm/year at x = 3%. This increase can be attributed to the aggregation of Sr-rich secondary phases, which become electrochemically active and act as local cathodes, thus accelerating the anodic dissolution of the Mg matrix through a mechanism of microstructural galvanic corrosion. Therefore, an optimal compositional balance is observed around x = 1% Sr, where the corrosion rate is minimal, while exceeding this threshold leads to accelerated degradation. Figure 8, Shows the pH variation recorded during corrosion rate evaluation tests for sample G5 over 72.

During the 72 h immersion test, a significant increase in the pH value of the SBF solution was observed, from the initial value of 7.4 (corresponding to physiological conditions) to values close to 10. This evolution is characteristic of magnesium-based systems, where the anodic dissolution of Mg in an aqueous environment leads to the formation of Mg^2+^ ions, according to the reaction:

Mg → Mg^2+^ + 2e^−^

The released electrons are consumed in the cathodic reaction:

2H_2_O + 2e^−^ → H_2_↑ + 2OH^−^

The generation of OH^−^ ions causes a progressive alkalinization of the medium, a phenomenon directly reflected by the increase in pH value. In the case of the Mg-Ca-Sr alloy, the presence of Ca and Sr can influence this process by forming corrosion products such as Mg(OH)_2_ hydroxides, which can deposit on the surface, partially contributing to its passivation, but not sufficiently to stabilize the pH.

An increase in pH above 9 indicates intense electrochemical activity, specific to the degradation of magnesium alloys in simulated biological environments. This behavior is relevant from a biomedical point of view, since high pH values can affect the local compatibility of resorbable implants, influencing cellular response and bone tissue formation.

**Figure 9:**
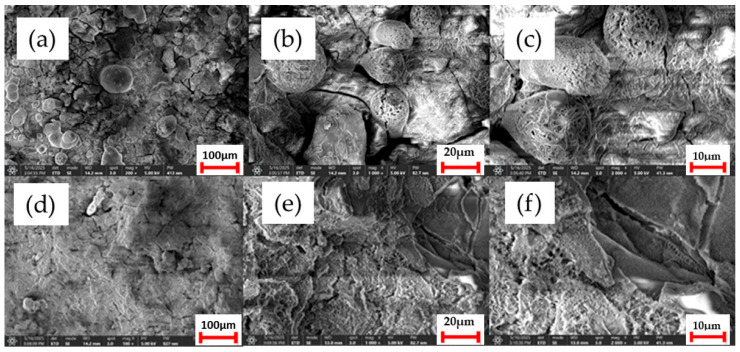
SEM images after corrosion for G1 alloy (**a**–**c**) and G5 alloy (**d**–**f**). Magnification by columns: ×200, ×1000, and ×2000.

Morphological changes on the sample surfaces before and after the corrosion tests, analyzed by scanning electron microscopy, are shown in Figure 9, and the chemical composition of the corrosion layers investigated using energy-dispersive spectroscopy (EDS) is presented in Table 5.

The morphological analysis of the Mg-0.5Ca-0.5Sr alloy surface, performed by scanning electron microscopy after exposure to a corrosive medium (SBF), reveals significant topographical changes associated with the electrochemical degradation process. The SEM images show a non-uniform surface, characterized by fine cracks and agglomerations of corrosion products.

Non-homogeneous deposits are observed across the entire surface, corresponding to typical corrosion products of magnesium-based systems, such as Mg(OH)_2_, as well as secondary compounds that include calcium and strontium, in the form of Ca(OH)_2_, Sr(OH)_2_, or calcium/strontium phosphates resulting from interactions with ions from the SBF solution (e.g., PO_4_^3−^, CO_3_^2−^).

To complement the morphological observations, surface chemical analysis was carried out by energy-dispersive spectroscopy (EDS). The obtained spectra indicate a majority composition of Mg, confirming the formation of magnesium hydroxide (Mg(OH)_2_) as the main corrosion product. Significant concentrations of Ca, P, and Sr were also detected, suggesting the presence of secondary compounds such as calcium phosphates (Ca_3_(PO_4_)_2_) and possible inclusions of Sr(OH)_2_ or strontium phosphates, originating from the interaction between the alloy and the ions available in the SBF (especially PO_4_^3−^ and Ca^2+^).

A notable observation is the apparent increase in the surface signal intensity associated with strontium in alloys containing higher Sr content. This trend may be attributed to the localized enrichment of Sr at the surface due to the selective nature of the corrosion process: magnesium, as the primary anodic component, preferentially dissolves, whereas Sr-containing intermetallic phases (e.g., Mg_17_Sr_2_) exhibit higher corrosion resistance and remain at the surface after matrix degradation. Given the semi-quantitative nature of EDS, such variations are interpreted as relative surface enrichment rather than exact compositional shifts.

SEM analysis of the Mg-0.5Ca-3Sr alloy surface, after 72 h of exposure in SBF solution, reveals an extensive corrosion layer with a porous and irregular morphology, characterized by agglomerations of solid compounds and areas of superficial exfoliation. Voluminous deposits of corrosion products are observed, particularly around intermetallic phases, indicating intense and non-uniform electrochemical activity.

The results obtained by energy-dispersive spectroscopy (EDS) confirm the presence of the characteristic elements on the surface: Mg, O, Ca and Sr, as well as noticeable amounts of P and Cl originating from the test solution. This suggests that these elements are actively involved in the formation of corrosion products. There is an increase in the relative content of Sr on the surface, from the initial value of 3% in the bulk alloy to 3.2% detected by EDS after corrosion. This local Sr enrichment can be explained by:

The selective dissolution of Mg during exposure leaves behind Sr-rich intermetallic phases (such as Mg_17_Sr_2_), which are relatively more stable in the corrosive environment, thus leading to an accumulation of residual Sr on the surface.

The local reprecipitation of Sr^2+^ ions is partially released into solution, but which, under alkaline pH conditions (pH > 9.5), can form poorly soluble compounds that redeposit as strontium salts or hydroxides.

This relative accumulation of strontium on the corroded surface can modify the local electrochemical reactivity, leading to an intensification of galvanic processes, since Sr-rich phases behave cathodically with respect to the Mg matrix. Thus, even if Sr contributes positively to microstructure refinement in the initial alloy, a high concentration (3%) can promote accelerated degradation in adjacent areas.

### 3.4. Cytocompatibility Evaluation

**Figure 10:**
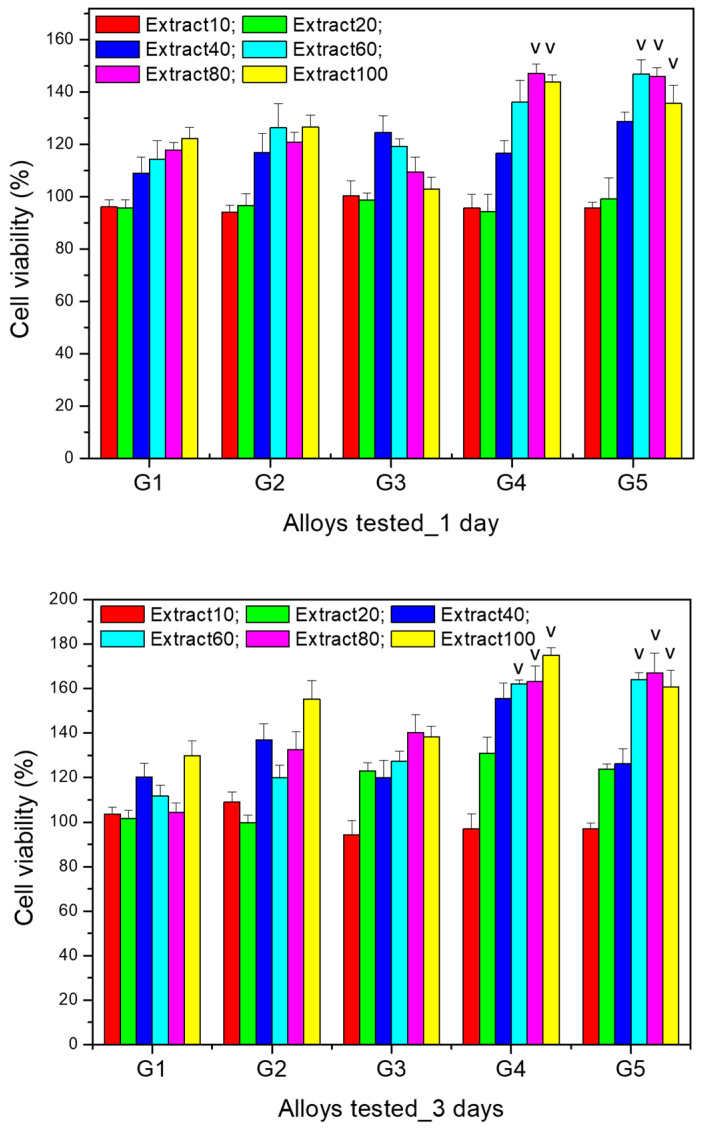
The results of the MTT assay for studying the viability of the MG63 osteoblasts co-incubated for 1 and 3 days with different extracts concentrations of the studied alloys in the Mg-0.5Ca-xSr system (i.e., G1 (0.5% Sr), G2 (0.1% Sr), G3 (1.5% Sr), G4 (2% Sr), G5 (3% Sr)); see the text for details. Cell viability is expressed as a percentage relative to the control well. (˅)—significant difference (*p* < 0.05) comparing the same extract of the different tested alloys.

The results of the cytocompatibility test for the comparative evaluation of the viability of MG63 cells after co-incubation (for 1 and 3 days) with extracts (i.e., extract 10%, 20%, 40%, 60%, 80%, 100%) of the Mg-0.5Ca-xSr studied alloys (i.e., G1 (0.5% Sr), G2 (0.1% Sr), G3 (1.5% Sr), G4 (2% Sr), G5 (3% Sr)) at various dilutions are presented in Figure 10.

The data resulted for cell viability tests performed after 24 h highlight that for the 10% extracts of all the alloys, there were no significantly different viability values (*p* > 0.05). A similar result was found for the 20% extract (*p* > 0.05). Moreover, after 24 h of culture, for the 60% extract the viability was significantly higher (*p* < 0.05) for alloy G5 (3% Sr) compared to the other alloys. In addition, for the 80% and 100% extracts, the viability was significantly higher (*p* < 0.05) for alloys G4 (2% Sr) and G5 (3% Sr) in comparison to the other studied alloys.

After 3 days of culture with the selected extracts (i.e., extract 10%, 20%, 40%, 60%, 80%, 100%) of the studied alloys, cell viability increased compared to the values recorded after one day and maintained the same trend of increasing viability with increasing extract concentration, with the increase being more significant starting from the 40% extract for all alloys. However, significantly higher values (*p* < 0.05) were recorded for alloys G4 (2% Sr) and G5 (3% Sr) for all the extracts studied, except for the 10% extract, for which the viability was not significantly different (*p* > 0.05) compared to the other studied alloys.

Regarding the cell viability profile recorded for the dilutions of the same alloy, there was an increase in cell viability after both 1 day and 3 days of culture, compared to the control wells (containing only cells cultured in medium without any extract), in a manner proportional to the extract concentration, the increase being more pronounced starting from the 40% extract. Moreover, the cell viability recorded for the 100% extract was significantly higher (*p* < 0.05) compared to the other dilutions studied; this was observed for all alloys after both 1 day and 3 days of culture.

The results of the cell viability tests highlight not only the non-cytotoxic nature of all Mg-0.5Ca-xSr studied alloys (i.e., G1 (0.5% Sr), G2 (0.1% Sr), G3 (1.5% Sr), G4 (2% Sr), G5 (3% Sr)), but rather a stimulatory effect on cell proliferation, since the MG63 cell viability cultured with the extracts of the studied alloys was statistically different (*p* < 0.05) and significantly higher (compared to the control well) even in the case of the 100% extracts.

These results can be attributed to the fact that the presence of Ca, Mg, and Sr ions had a favorable effect on cell viability and a stimulatory effect on cell proliferation, despite the corrosion results showed a higher degree of corrosion for alloys’ samples having a Sr content ≥ 1.5%.

**Figure 11:**
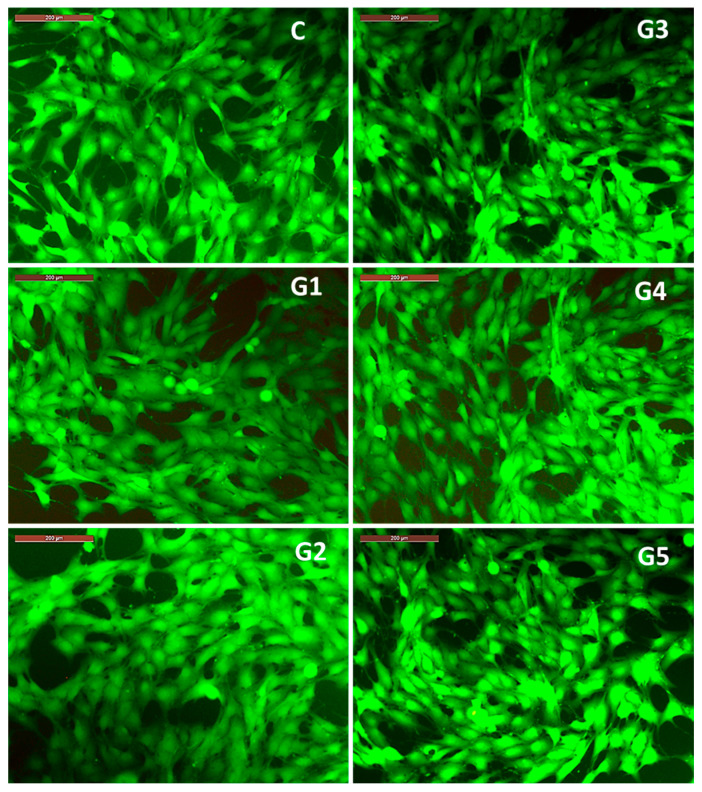
Images captured with the fluorescence microscope highlighting the morphology of MG63 cells co-incubated for 3 days with 100% extracts of each alloy in the studied Mg-0.5Ca-xSr system (i.e., **G1** (0.5% Sr), **G2** (0.1% Sr), **G3** (1.5% Sr), **G4** (2% Sr), **G5** (3% Sr)), and the control well (**C**). Staining with Calcein-AM. Scale bar 200 µm.

The results regarding cell viability correlate with the observations on the morphology of MG63 osteoblasts (Figure 11) co-incubated for 3 days with 100% extracts of the studied alloys. Thus, it is observed that the cell morphology of MG63 cell cultured with 100% extracts is similar to that recorded in the control well (containing only cells cultured in culture medium, without extract resulting from alloy degradation), showing a polygonal morphology with broad cytoplasmic processes of the lamellipodia or filopodia type (Figure 11). The observed cell morphology, correlated with the significant cell viability profile recorded after 3 days of co-incubation of MG63 cells with the 100% extracts, highlights the fact that the microenvironment in the wells (with different and increased concentrations of Ca, Mg and Sr ions compared to the culture medium) did not negatively influence either cell viability or cell morphology, bearing in mind that accumulation of ions, even with physiological roles, above a certain threshold can have an inhibitory or cytotoxic effect. In this case, the favorable effect of the ions released from the degradation of the studied alloys can be explained by the fact that calcium ions are associated with a structural role related to the reorganization of cytoskeletal elements [42,43], magnesium ions have a role in regulating the expression of genes and proteins involved in cell growth and osteogenesis [44], and strontium is known to have an osteogenic role, stimulating cell proliferation and differentiation [45].

It is worth mentioning that the degradation process of magnesium-based alloys is influenced by the microenvironmental conditions; since in vivo biodegradation processes occur in a microenvironment governed by active transport phenomena, it is to be expected that histological analysis will show the favorable alloys integration in the bone tissues.

### 3.5. Histopathological Evaluation

**Figure 12:**
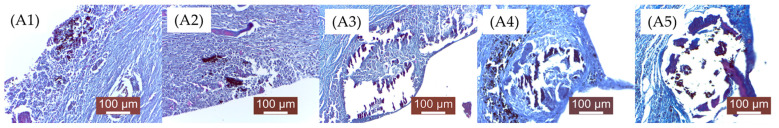
Histopathological examination micrographs for samples (**A1**—**A5**) (i.e., Mg-0.5Ca-xSr alloys containing 0.5, 0.1, 1.5, 2, 3% Sr) after 2 weeks of implantation (see the text for details).

**Figure 13:**
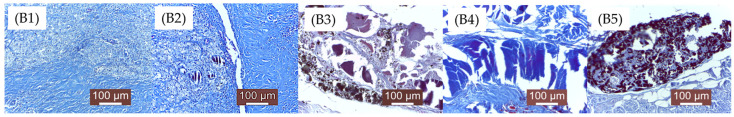
Histological aspects for samples (**B1**–**B5**) (i.e., Mg-0.5Ca-xSr alloys containing 0.5, 0.1, 1.5, 2, 3% Sr) after 4 weeks of implantation (see the text for details).

**Figure 14:**
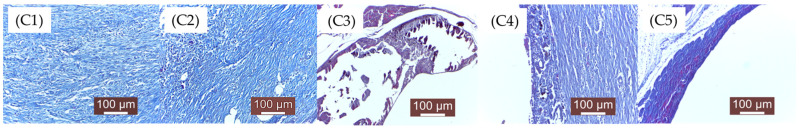
Histopathological examination micrographs for samples (**C1**–**C5**) (i.e., Mg-0.5Ca-xSr alloys containing 0.5, 0.1, 1.5, 2, 3% Sr) after 12 weeks of implantation (see the text for details).

As mentioned in the Materials and Methods chapter, the groups were numbered as follows: A—2 weeks (A1, A2, A3, A4, A5), Figure 12; B—4 weeks (B1, B2, B3, B4, B5), Figure 13; C—12 weeks (C1, C2, C3, C4, C5), Figure 14. 1, 2, 3, 4, 5—the implanted materials with increasing concentrations of Sr.

Through qualitative histopathological examination, the tissue biocompatibility of the implanted material was comparatively evaluated in all experimental groups. For this purpose, the evolution of the local post-implant inflammatory process, the degree of resorption/degradation of the implanted material, and the scarring process resulting after material absorption (the appearance of the newly formed connective tissue that replaced the material) were assessed.

A1—Subacute-chronic inflammatory process, characterized by a moderate leukocyte infiltration, mainly macrophages and neutrophils. The implanted material appears as small, dispersed enclaves, surrounded by young connective fibers. Numerous fibroblasts and collagen fibers are observed, arranged as peri-implant fibrous trabeculae, as well as, very importantly, newly formed blood capillaries that provide nutritional support to the newly formed connective tissue. In some areas, the presence of leukocytes still persists. Near the implantation site, gas bubbles are visible, moderately separating local structures. Outside the area where the material was placed, a fairly consistent fibrous capsule has formed, rich in newly formed blood capillaries, aiming to separate the material from the adjacent muscle fibers.

A2—Compared to concentration 1, there are no significant modifications. The implanted material was disintegrated into smaller islands, surrounded by a moderate inflammatory reaction, characterized by a moderate leukocyte influx (macrophages, neutrophils, lymphocytes) and young connective tissue. Gas bubbles (H_2_), probably resulting from material degradation, were also noted. Young connective tissue, fibroblasts, rare macrophages and lymphocytes, and newly formed blood capillaries are observed.

A3—Larger enclaves of implanted material, surrounded by less connective tissue; inflammation is moderate, represented by activated mesenchymal cells. The interstitial tissue is separated by gas (H_2_) resulting from tissue degradation of the material. The connective capsule developed around the implant is less pronounced than the previous groups (A1, A2). The implanted material is incompletely resorbed, surrounded by newly formed connective tissue.

A4—The implanted material still persists in a significant amount, surrounded by a moderate inflammatory reaction and a newly formed collagen fibers capsule. The aspect is similar to group A3. Peripheral mesenchymal reaction represented by macrophage infiltration and loose, young connective tissue; rare newly formed blood capillaries. A large amount of implanted material is surrounded by a moderate inflammatory reaction and peripheral neogenesis of connective tissue. The quantities of implanted material are higher than in the previous groups.

A5—Large amount of remaining implanted material, compared to previous batches, delimited by a moderate inflammatory reaction and neogenesis of peripheral connective tissue.

B1. At 4 weeks, the implanted material is almost resorbed, being replaced by significant connective tissue in the process of maturation and numerous newly formed blood capillaries. A consistent connective capsule is observed, in the process of organization, with rare lymphocytes and histiocytes. Significant biodegradation of the implanted material is noted.

B2. Small dispersed areas of material are delimited by newly formed connective tissue. The inflammatory phenomenon is moderate. In some areas, the presence of the material and an inflammatory reaction around it are still observed.

B3. The implanted material still persists, being surrounded by a non-uniform, moderate connective reaction. Compared to the previous groups (B1, B2), degradation of the material was less pronounced, with the material still present at the tissue level.

B4. Areas of material are still present, surrounded by a fairly consistent collagen fibers capsule. The inflammatory reaction is represented by macrophages, rare lymphocytes, and histiocytes around the material.

B5. The implanted material is not degraded, still persisting. It is surrounded by a peripheral connective capsule. Its resorption was slower than in the previous groups.

C1. The implanted material is completely resorbed. The connective tissue formed in the implanted area is well organized, still maturing, rich in fibroblasts and collagen fibers. The inflammatory infiltrate is absent. The implanted material is no longer detectable, having been completely degraded. The implanted area is represented by almost mature, well-structured connective tissue with well-oriented collagen fibers and rare fibroblasts. Numerous newly formed blood vessels are present, providing adequate nutrition of the area.

C2. The implantation area is characterized by the presence of mature connective tissue, with denser areas richer in fibers and areas of looser (less dense) connective tissue and fibroblasts. Numerous newly formed blood vessels are also observed.

C3. There are still tissue areas in which the implanted material is present in significant amounts/large blocks. Local inflammation is reduced, and scar connective tissue is present in moderate quantities. Persistent implanted material is surrounded by a collagen fibers capsule separated by gas (H_2_) resulting from its tissue resorption.

C4. The implanted material is still present, with a peripheral connective fibers reaction. The same dissociation of the connective tissue with gas resulting from the tissue reaction to the material is observed. The situation is similar to C3. Small areas with implanted material are still visible on histological examination. Degradation was slower compared to the previous groups (C1, C2, C3).

C5. A distended connective tissue capsule was observed around the area of gas (H_2_) accumulation resulting from the biodegradation of the material. The inflammatory reaction is absent. The material is also absent.

## 4. Conclusions

1. Microstructural analyses confirm the formation of uniform structures, without microcracks, inhomogeneities, or other defects. Additionally, a refined microstructure was obtained, attributed to the presence of Ca and the compounds Mg_2_Ca, MgSr, Mg_2_Sr, and Mg_17_Sr_2_, which improve microstructural refinement.

2. Through the determination of electrochemical corrosion behavior, increasing the Sr content in Mg-0.5Ca-xSr alloys from 0.5% to 3% leads to a non-linear evolution in corrosion behavior. At low concentrations (0.5–1%), Sr slightly improves corrosion resistance due to the refined microstructure. However, exceeding the threshold of ~1.5% Sr leads to the formation of cathodic intermetallic phases (e.g., Mg_17_Sr_2_), which facilitate galvanic corrosion. Thus, the corrosion rate increases significantly at x ≥ 2%, indicating that this content becomes detrimental for applications where corrosion resistance is essential.

3. Following the cytocompatibility evaluation tests, no cytotoxic character was observed, but rather a stimulation of proliferation and cell viability (in the case of all the studied alloys), both after 1 day and after 3 days of culture, compared to the control well, in a manner proportional to the extract concentration, with the increase being more pronounced starting from the 40% extract. The cell viability recorded for the 100% extract was significantly higher (*p* < 0.05) compared to the other dilutions studied, a finding observed for all alloys both after 1 day and after 3 days of culture. Significantly higher cell viability values (*p* < 0.05) after 3 days were recorded for alloys G4 (2% Sr) and G5 (3% Sr) for all the extracts studied.

4. Following in vivo tests evaluating the tissue biocompatibility of the alloys, materials G1 and G2 showed a faster degradation compared to G3, G4, and G5 materials, which were still present even 12 weeks after implantation. In all the examined groups, the inflammatory response was directly proportional and persistent with the presence of material at the tissue level. Where the material was resorbed/degraded, the local inflammatory response was reduced or absent, and the fibrous tissue was more consistent and better organized.

5. For all the tested materials, intratissue gas (H_2_) release was noted in the areas adjacent to the implant, which dissociated local structures. In all the groups, this diminished as the material was completely resorbed and disappeared shortly after the total biodegradation of the implanted materials.

6. The gradual resorption of the material was directly proportional to the neogenesis of connective tissue (newly formed connective tissue) at the implantation site. The faster the resorption, the more intense the connective neogenesis.

7. In groups 1 and 2, at the end of the experiment, a well-organized scar connective tissue rich in newly formed capillaries was observed.

## Figures and Tables

**Table 1:** Types of biomaterials [2,3,4].

Types of Biomaterials	Advantages	Disadvantages	Examples
**Metals and Metallic Alloys**			
Ti and Ti alloys, stainless steels, Co-Cr alloys, Au alloys, Hg-Ag-Sn amalgams, platinum and Pt-Ir alloys, etc.	High mechanical strength, formability by deformation, toughness	Corrode in the human body environment	Joint replacements, bone plates and screws, dental root implants
**Polymers**			
Polyesters, polyurethanes, polypropylene, hydrogels, polyamides, polyvinyl chloride, PET, PTFE, silicones, etc.	Easily manufactured, resilient, relatively low cost, property adaptability	Deform over time, may degrade in the biological environment, insufficient mechanical strength	Sutures, blood vessels, ears, nose, other soft tissues, hip joints, dental restorations, ophthalmology
**Ceramics and Glasses**			
Alumina, calcium phosphates, carbon, bioactive glasses, porcelains, zirconia, etc.	High compressive strength, bioactivity, chemical inertness	Brittleness, difficult to manufacture	Joint replacements, dental implants, dental restorations, bone tissue repair and regeneration
**Composites**			
Acrylic resin/glass, polymer/carbon, carbon/carbon	Customizable properties and sizes depending on application	Difficult to manufacture	Dental restorations, joint replacements

**Table 2:** Chemical composition of simulated body fluid (SBF) solution [14].

Nr.Crt.	Reagent	Amount
1	NaCl	8.035 g
2	NaHCO_3_	0.355 g
3	KCl	0.225 g
4	K_2_HPO_4_ · 3H_2_O	0.231 g
5	MgC_l2_ · 6H_2_O	0.311 g
6	1.0 M-HCl	39 mL
7	CaC_l2_	0.292 g
8	Na_2_SO_4_	0.072 g
9	Tris	6.118 g
10	1.0 M-HCl	0–5 mL

**Table 3:** Electrochemical corrosion parameters.

Sample	E(I = 0) (mV)	j_corr_ (mA/cm)	Rp (ohm·cm)	v_corr_ (mm/year)	–β_c_ (mV/dec)	β_a_ (mV/dec)
G1	−1976	0.4748	54.49	10.95	159	164
G2	1805	0.1981	99.18	4.56	102.8	118
G3	1896	0.3931	131.77	9.06	372.2	291.8
G4	2020	0.5170	97.41	11.92	327.8	331.2
G5	1926	0.6387	55.67	14.73	265.1	163.7

**Table 4:** Corrosion rates determined by each mass loss of samples subjected to immersion tests (in SBF) and subsequent cleaning in the ultrasonic bath (in ethylic alcohol). (Immersion time—for 3 days.)

Samples	G1	G2	G3	G4	G5	G5 pH
Initial mass (g)	0.2817	0.3117	0.2262	0.3515	0.2172	0.2621
Mass after immersion (g)	0.2725 (−0.0092)	0.3019 (−0.0098)	0.2140 (−0.0122)	0.3422 (−0.0093)	0.1953 (−0.0219)	0
Mass after ultrasonic cleaning (g)	0.2677 (−0.014)	0.2979 (−0.0138)	0.2128 (−0.0134)	0.3361 (−0.0154)	0.1908 (−0.0264)	
Aria	1.25 cm^2^	1.38 cm^2^	1.25 cm^2^	1.25 cm^2^	1.55 cm^2^	1.55 cm^2^
CR (mm/year)	7.83	6.98	7.47	8.57	11.81	

Standard Deviation: ±0.0001 g; $CR=\frac{8.76\times 10^{4}W}{At\rho }$, [41]; CR = corrosion rate [mm/y]; (w = mass loss [g], A = sample area [cm^2^], t = time [h] and ρ = density [g/cm^3^]).

**Table 5:** Chemical composition—EDS.

Element	G1 [%]	G5 [%]
Na	0.0	0.0
Mg	75.0	57.6
P	5.4	0.9
Cl	16.2	36.5
Ca	2.7	1.8
Sr	0.7	3.2

## Data Availability

The original contributions presented in this study are included in the article. Further inquiries can be directed to the corresponding authors.
